# A Comparative Study on Nickel Binding to Hpn-like Polypeptides from Two *Helicobacter pylori* Strains

**DOI:** 10.3390/ijms222413210

**Published:** 2021-12-08

**Authors:** Danuta Witkowska, Agnieszka Szebesczyk, Joanna Wątły, Michał Braczkowski, Magdalena Rowińska-Żyrek

**Affiliations:** 1 Institute of Health Sciences, University of Opole, Katowicka 68, 45-060 Opole, Poland; agnieszka.szebesczyk@uni.opole.pl; 2 Faculty of Chemistry, University of Wroclaw, F. Joliot-Curie 14, 50-383 Wroclaw, Poland; joanna.watly@chem.uni.wroc.pl (J.W.); magdalena.rowinska-zyrek@chem.uni.wroc.pl (M.R.-Ż.); 3 Institute of Medical Sciences, University of Opole, Oleska 48, 45-052 Opole, Poland; michal.braczkowski@uni.opole.pl

**Keywords:** nickel binding, *H. pylori*, Hpn-like, histidine-rich, glutamine-rich, ATCUN motif

## Abstract

Combined potentiometric titration and isothermal titration calorimetry (ITC) methods were used to study the interactions of nickel(II) ions with the N-terminal fragments and histidine-rich fragments of Hpn-like protein from two *Helicobacter pylori* strains (11637 and 26695). The ITC measurements were performed at various temperatures and buffers in order to extract proton-independent reaction enthalpies of nickel binding to each of the studied protein fragments. We bring up the problem of ITC results of nickel binding to the Hpn-like protein being not always compatible with those from potentiometry and MS regarding the stoichiometry and affinity. The roles of the ATCUN motif and multiple His and Gln residues in Ni(II) binding are discussed. The results provided the possibility to compare the Ni(II) binding properties between N-terminal and histidine-rich part of Hpn-like protein and between N-terminal parts of two Hpn-like strains, which differ mainly in the number of glutamine residues.

## 1. Introduction

The Hpn-like histidine (H) and glutamine (Q)-rich protein play at least two roles in *Helicobacter pylori* (*H. pylori*). This protein is transcriptionally activated in the presence of nickel by the nickel sensor *NikR* and appears to compete with the Ni-dependent urease maturation machinery under low-nickel availability. Nickel is released from Hpnl at acidic pH (the nickel storage and sequestering role). However, when the external nickel levels reach toxic limits, Hpn-like protein can render nickel tolerance to *H. pylori* [1,2].

Both roles are important for urease and hydrogenase enzymes activity and for *H. pylori* survival in the human stomach. *H. pylori* infections can lead to serious health consequences such as chronic gastritis, peptic ulcer disease (PUD), gastric mucosa-associated lymphoid tissue (MALT) lymphoma, and gastric cancer [3]. Some researchers suggest potential mechanisms linking *H. pylori* infection also with iron deficiency anemia [4]. There are other nickel-dependent enteric pathogens, such as *Salmonella*, *Proteus, Klebsiella*, *Shigella*, and *Yersinia* species, to name a few, which are responsible for millions of cases of illness annually [2,5].

Over the last years, the multi-drug resistance of the *H. pylori* and *Enterobacteriaceae* family has arisen, which drives researchers to search for new ways to disable these bacteria [6,7]. One of such possibilities can be nickel chelation therapy because nickel ions are required for most enteric pathogens but not for human enzymes. Ni(II)-specific chelator could inhibit the activity of two important bacterial enzymes, hydrogenase, and urease. However, it needs to be safe for human health [7].

Many studies are also focused on nickel-accessory proteins, an example of which are Hpn-like proteins [8,9,10].

Studies of H. Sun and co-workers show that Hpn-like protein binds two Ni(II) ions per one molecule and that both nickel-binding sites are localized in the His-rich domain (residues 21 to 43) [11]. That same stoichiometry was shown for Cu(II), Co(II), and Zn(II) binding. The isothermal titration calorimetry (ITC) measurements in that work were performed in tris(hydroxymethyl)aminomethane (TRIS) buffer.

Generally, TRIS buffer is not recommended for studies on metal ions interactions, as it binds divalent metal ions with considerable stability constants, which can lead to misinterpretation [12].

Moreover, Zn(Tris)^2+^ and Co(Tris)^2+^ complexes (log K = 1.94 and log K = 1.73, respectively) were shown to be about 100-fold less stable than Cu(Tris)^2+^ (log K = 4.05), and approximately 10-fold less stable than Ni(Tris)^2+^ (log K = 2.74) [12]. On the other hand, the formation of well-defined Ni(II)-buffer complexes prevents metal hydrolysis reactions and allows the metal-buffer interactions to be precisely subtracted from K_ITC_ and ΔH_ITC._ To be able to compare the ITC results and obtain unconditional data, appropriate post-hoc analysis is needed [13,14].

Our previous potentiometric and spectroscopic studies showed that the glutamine-rich N-terminal part of Hpn-like (strain 26695) binds nickel(II) and copper(II) ions with higher affinity than the similar motif of Hpn protein (with only one glutamine residue in the sequence), and even than the N-terminal part of human albumin [15], an important physiological transporter of the divalent metal ions in the bloodstream [16].

**Figure 1:**
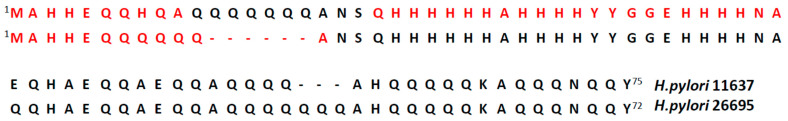
Amino acid sequence of *H. pylori* Hpnl protein (strains 11637 and 26695). The sequences used as peptides under this study are shown in red.

All this taken together prompted an idea to design peptide analogs of N-terminal parts of *H. pylori* Hpn-like protein (strain 11637 and 26695) and of its histidine-rich motif, and to compare their binding ability toward nickel(II) ions by using isothermal titration calorimetry (ITC), potentiometry, and MS techniques. Hpnl proteins from both chosen strains have the same sequence of the histidine-rich motif and different N-terminal regions (Figure 1).

In the last of the designed peptide analogs (^21^QHHHHHHAAHHHYYGGEHHHHNA^43^), the His residue in the 29th position (the 11637 strain in Figure 1) was mutated to alanine (A) to check if this residue is crucial for Ni(II) binding to the Hpn-like protein, as it had been suggested before [11].

**Figure 2:**
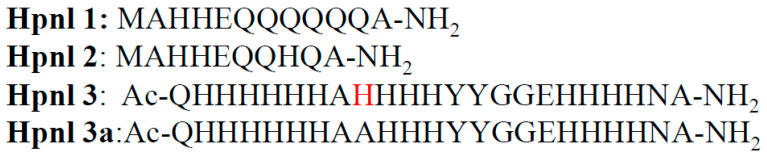
Amino acid sequences of Hpnl1, Hpnl2, Hpnl3, and Hpnl3a peptides. The His residue mutated to Ala is shown in red.

The thermodynamic analysis of nickel binding to these four designed peptides (shown in Figure 2) was carried out by the ITC technique, in three different buffers (TRIS, HEPES, and MOPS; pH 7.40), at two temperatures (25 and 37 °C), and the post-hoc analysis of ITC results has been performed.

HEPES (2-[4-(2-hydroxyethyl)piperazin-1-yl]ethanesulfonic acid) buffer is described in the literature as a non-complexing buffer for Ni(II) ions [17], and similarly MOPS (3-(N-morpholino)propanesulfonic acid), at least within the buffering range of pH 6.50–7.90 [18]. These buffers were chosen for ITC experiments, as well as 20 mM TRIS-HCl buffer + 500 mM NaCl, to repeat the exact conditions of the mentioned above work of Sun et al. [11].

## 2. Results and Discussion

**Figure 3:**
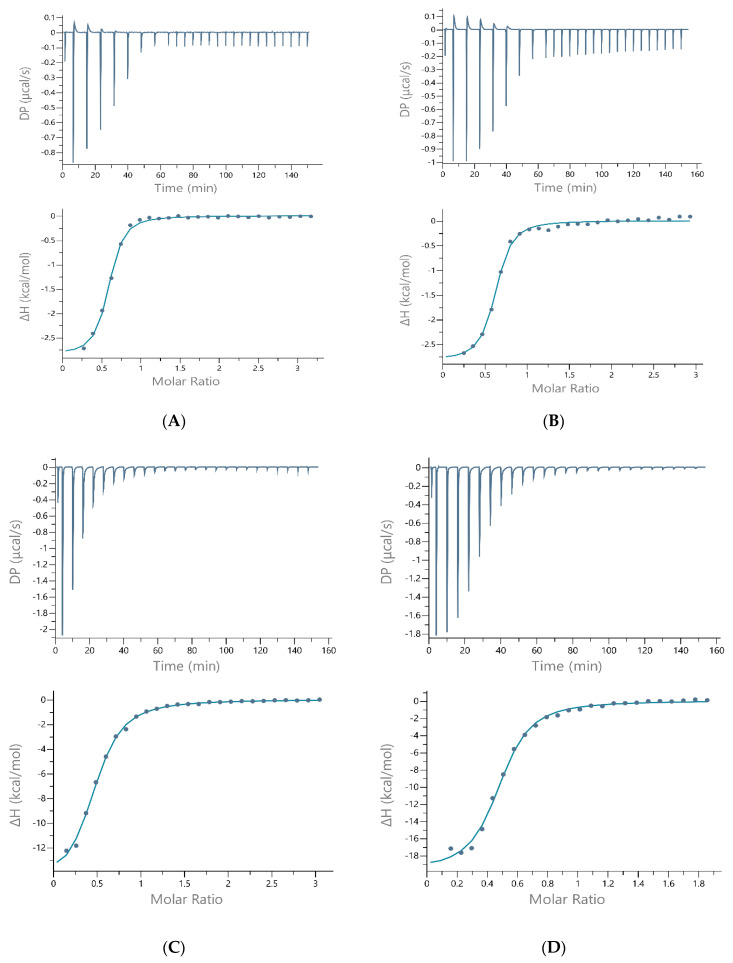
The best-fit ITC data for the 37 °C titration of 1–2 mM Ni(II) into (**A**) Hpnl1, (**B**) Hpnl2, (**C**) Hpnl3, (**D**) Hpnl3a peptides in 50 mM MOPS + 50 mM KCl, pH 7.40. The peptide concentration was within the range of 100–133 µM.

The results of ITC measurements at 37 °C are shown in Table 1 and in Figure 3, Figures S1 and S2. The data (stoichiometry, affinity, enthalpy, and entropy of nickel-peptide binding) must be analyzed for each buffer separately, as these buffers have a different enthalpy of reaction. Moreover, one of them (TRIS buffer) was shown to bind Cu(II) and Ni (II). Indeed, a significant difference in the binding enthalpy was found when the buffer was changed to MOPS and HEPES, indicating that TRIS is interacting with the Ni(II)-peptide complex. Most probably, because of its protonation site, a primary amine that is more accessible to coordinate to metal than protonation sites of MOPS and HEPES [19].

It has been shown previously that the unprotected N-terminal site with the His residue at the first position can compete with poly-imidazole coordination sites for divalent metal ions [20]. Furthermore, in a narrow range of pH, Ni(II) and Cu(II) binding to His(6)-tag (poly-imidazole sequence) can even compete with the albumin-like binding (in which the metal ion is bound to the imidazole of His-3, to the N-terminal amine nitrogen and two amide nitrogens situated between the N-terminus and His-3) [21].

Two of the peptides in this study have a free N-terminal amino group and His residue in the third position (ATCUN-binding motif). Potentiometry shows that the peptides with the ATCUN motif bind Cu(II) and Ni(II) in a 1:1 stoichiometry, in an “albumin-like mode” (through the N-terminal NH_2_, the imidazole of the His residue in the third position, and two amide nitrogens in between) [15,22]. Surprisingly, the ITC results in this work (measurements performed at 37 °C) reveal that the stoichiometry is 1:2 for nickel binding to all peptides in all buffers (N is approximately 0.5, as shown in Table 1). That can suggest two ligand binding to one metal ion.

The next finding that can be drawn from the ITC data (analyzing the results in Table 1 for each buffer separately) is that the affinity of Hpnl1 and Hpnl2 for Ni(II) ions is very similar: K_d_ is within the range of 2–3.20 µM. The affinity of Hpnl3 (His-rich sequence of Hpnl protein) for Ni(II) ions is slightly lower (K_d_ is equal to 4.64; 7.8 and 8.3 µM for measurements performed in TRIS, HEPES, and MOPS buffers, respectively).

The His29A mutation led to the formation of slightly more stable nickel complexes (K_ds_ of Ni(II)-Hpnl3a are lower than K_ds_ of Ni(II)-Hpnl3 complexes, comparing the measurements in each buffer separately), suggesting that this residue is not crucial for Ni(II) binding to the analog of His-rich part of *H. pylori* Hpn protein. That same tendency can be observed for measurements taken at 25 °C (Table 2). The Ni(II)-Hpnl3a binding is also more enthalpy-driven than nickel binding to Hpnl3. The favorable increase in enthalpy can be correlated with the interactions resulting from metal-mediated folding.

Also, the entropic penalty is bigger for Hpnl3a binding, which can be connected with the higher hydrophobicity of Ala than His residue.

**Figure 4:**
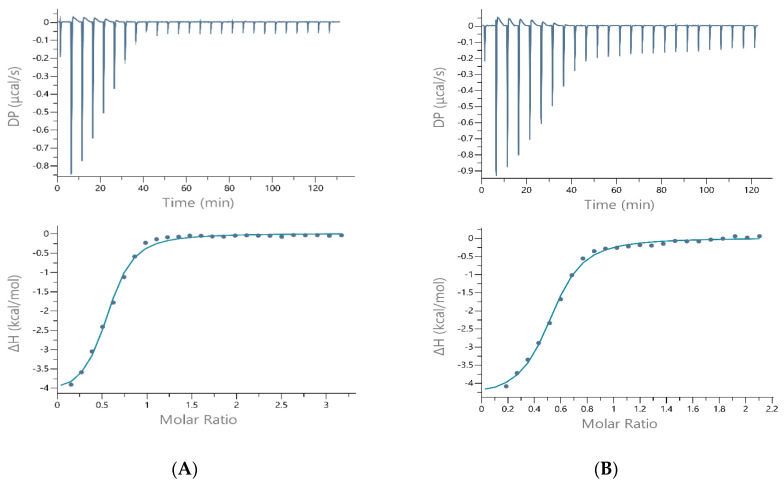

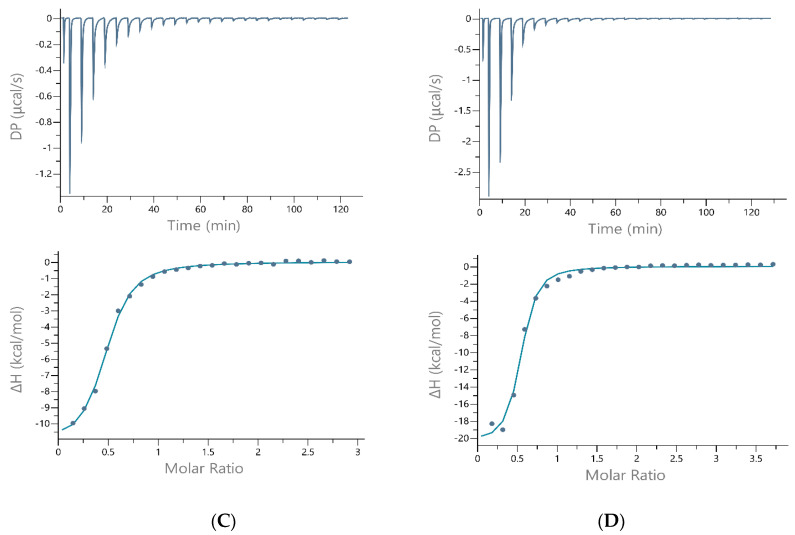
The best-fit ITC data for the 25 °C titration of 1.5–2 mM Ni(II) into (**A**) Hpnl1, (**B**) Hpnl2, (**C**) Hpnl3, (**D**) Hpnl3a peptides in 50 mM MOPS + 50 mM KCl, pH 7.40. The peptide concentration was within the range of 105–133 µM.

ITC traces show that the reaction is exothermic in all buffers and for all peptides; however, a biphasic injection profile for Ni(II) binding to Hpnl1 and Hpnl2 peptides can be observed for measurements performed in HEPES and MOPS buffers (Figure 3, Figure 4 and Figure S4).

Our results are not in agreement with previous studies on the Hpn-like protein [11]. There can be at least a couple of reasons for this phenomenon regarding the stoichiometry: (1) present studies are performed on Hpn-like fragments, not on the whole protein; (2) best-fit ITC values in the previous work [11] show that two Ni(II) ions bind to one Hpnl protein, which can also mean a four-metal ion binding to one homo-dimer of Hpnl.

On the other hand, there is one situation in our study where a 1:1 stoichiometry has been shown (Table 2). The assay was repeated a few times, proving the strange behavior of poly-histidine fragments (Hpnl 3 and 3a). We suppose that in the above-mentioned case, the arrangement of the factors (temperature, TRIS buffer comprising 500 mM of NaCl) prevented the dimer formation, and each peptide molecule “was able” to bind one Ni ion.

As ITC measures the *net* thermodynamics of binding, therefore buffer competition with the peptide for the metal and proton competition with the metal for the peptide must be considered in order to find the *condition-independent* stability constant (K) and enthalpy of reaction (ΔH°) [13,23].

To perform such post-hoc analysis, ITC studies have also been performed at 25 °C, in three buffers, supported by the potentiometric measurements and mathematical models. The condition-dependent results, before this post-hoc analysis, are shown in Table 2. Figure 4 shows the ITC traces of the nickel(II) titration to all four peptides in the MOPS buffer. Similar traces of Ni(II) to each peptide titration in TRIS and HEPES buffers are shown in Figures S3 and S4, respectively.

K_ITC_ means the apparent equilibrium constant including all coupled equilibria within the sample cell. The condition-independent equilibrium constant (K_MP_) can be quantified by Equation (1).

K_MP_ = K_ITC_ × α_buffer_ × α_proton_ (1)

The α_buffer_ accounts for buffer competition with the peptide for Ni(II) ion and was quantified by Equation (2) for TRIS buffer. As stated above, the condition-independent binding constant of Ni (II) to TRIS is known (log K = 2.74) and was included in the data analysis.

α_buffer_ = 1 + K_NiB_[TRIS] (2)

The α_proton_ accounts for Ni(II) competition with protons for the interacting amino acids and can be quantified by Equation (3), which is a function of the pH and pK_a_s of the peptide.

α_proton_ = 1 + K_HP_ [H^+^] + ß_2,_ PH_2_^2+^ [H^+^]^2^ + … (3)

Protonation constants of the peptides studied within this work were determined by potentiometric measurements at a pH range of 2–11. The results are collected in Table 3 and remain in suitable agreement with previous studies of similar systems [15].

The highest values (7.53 and 7.58) are in suitable agreement with literature data for the deprotonation of amino groups. Log K of 4.07 and 3.95 could be assigned to carboxylic groups of glutamic acid from Hpnl1 and Hpnl2, respectively. Other values (6.54 and 5.97 for Hpnl1, and 6.77, 6.28, and 5.73 for Hpnl2) characterize deprotonation of imidazole nitrogen of histidine residues. The potentiometric titrations were performed for the Ni(II)-to-ligand ratio 1:1 and 1:2 (Table S1 and Figures S5–S8). In all cases (for Hpnl1 and Hpnl2 peptides), the stoichiometry was shown to be 1:1. Potentiometric titrations of poly-histidine peptides (Hpnl3 and Hpnl3a) were performed, but due to the superimposition of multiple values of deprotonation constants, the calculations failed. Additionally, the buffer solutions of His-rich peptides (Hpnl3 and Hpnl3a) after some time became slightly cloudy, which suggests the intermolecular association involved in the formation of native oligomers. Sun et al. estimated before, using gel-filtration chromatography, that the molecular mass of the whole Hpnl protein was 201 kDa, which corresponds to a 22(±1)-mer [9].

Because of this, we could determine the independent thermodynamic values (K, ΔH) only for the Hpnl1 and Hpnl2 peptides. Using appropriate formulas (Equations (1)–(3)), the pH- and buffer-independent Ni(II) peptide binding constants have been quantified for Hpnl1 and Hpnl2 peptides as K = 3.93 × 10^6^ (K_d_ = 0.28 µM) and K = 6.44 × 10^6^ (K_d_ = 0.15 µM), respectively. In the scope of these studies (which were performed at pH 7.4), only Ni(II) competition with protons for the N-terminal amines was quantified to obtain α_proton._ The data show suitable agreement with the equilibrium dialysis data, which displayed a very similar affinity of Ni(II) ions for the Hpnl protein (K_d_ of 3.8 ± 0.2 µM) [9].

The competition plot for Ni-complexes with these peptides (based on potentiometric data and showing a hypothetical situation in which equimolar amounts of ligands and metal are present in solution; Figure S9) reveals that in potentiometric studies, MAHHEQQQQQQA-NH_2_ (Hpnl1) binds Ni(II) ions forming more thermodynamically stable complexes than the MAHHEQQHQA-NH_2_ (Hpnl2) peptide. Our previous potentiometric and spectroscopic studies on similar sequences having with the -XXH- N-terminal motif proved that these peptides bind Ni(II) and Cu(II) in the albumin-like mode [15,24].

On the other hand, calorimetric measurements within this study show that the affinity of nickel ions binding to the Hpnl2 peptide is slightly higher than that to the Hpnl1 peptide. There is a possibility that in this kind of complex (two molecules of peptide binding one metal ion), nickel(II) is bound only to His residues, and the poly-Q sequence (present in Hpnl1 peptide) does not support the binding.

**Figure 5:**
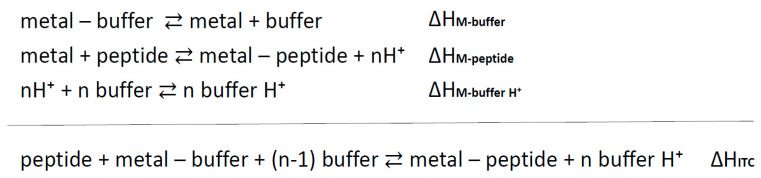
Scheme showing the enthalpies contributing to the ΔH_ITC_.

Performing the same titration in multiple buffers at the same pH provides the possibility to quantify the number of protons that are displaced from the peptide/protein upon the metal ion binding [13,25]. The contributing enthalpies (as shown in Figure 5) are known from the literature or can be determined using similar calorimetric experiments.

The proton transfer, which accompanies metal binding at a given pH, can be determined experimentally by the buffer protonation contribution to ΔH_ITC_. Equation (4) indicates the relationship between ΔH_ITC_ and ΔH°_HB_ [13].

ΔH_ITC_ + ΔH°_MB_ = n_H+_ (ΔH°_HB_ − ΔH°_HP_) + ΔH°_MP_ (4)

The n_H+_ in Equation (4) stands for the slope, which quantifies the number of protons binding to the buffer, for data collected at the same pH in two or more buffers with different protonation enthalpies.

The enthalpies of the metal-buffer complexation reactions (ΔH°_MB_) were calculated by displacement titration using ethylenediaminetetraacetic acid (EDTA) as a strong binding, competitive ligand [26]. In these measurements, a weak ligand (buffer TRIS, HEPES, or MOPS) was replaced by a strong one in the coordination sphere of the central ion. The enthalpy of Ni(II)-EDTA (−7.5 kcal/mol), as well as the EDTA-deprotonation (H1: −5.2 kcal/mol, H2: −3.8 kcal/mol), and buffer-protonation enthalpies (TRIS: −11.58 kcal/mol; HEPES: −5.04 kcal/mol; and MOPS: −5.30 kcal/mol) have been taken from the literature [27]. The Ni(II)-buffer interaction enthalpy has been solved using Equation (5) after rearrangement. The ITC traces, as well as the average enthalpies of Ni(II) to EDTA titration in each buffer, are shown in Figure S10.

ΔH_ITC_ = (−ΔH°_MB_) − (0.99 × ΔH_LH_) − (0.05 × ΔH_LH2_) + (ΔH°_ML_) + (1.04 × ΔH°_BH_) (5)

The quantified enthalpies (ΔH°_MB_) are listed below:

- for Ni(II)-TRIS interaction: 3.2 kcal/mol;
- for Ni(II)-HEPES interaction: 0.54 kcal/mol;
- for Ni(II)-MOPS interaction: 2.07 kcal/mol.

**Figure 6:**
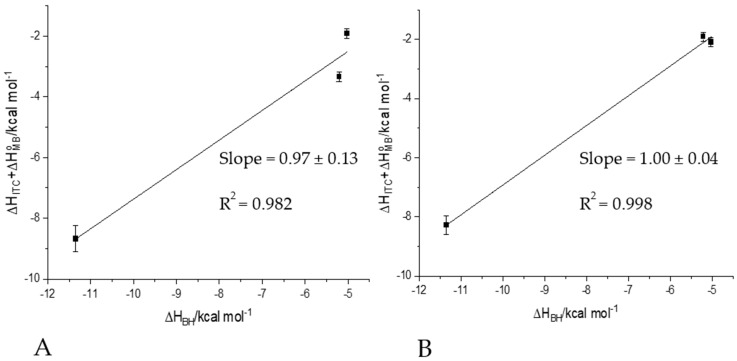
Protonation plot showing that the buffer is protonated (**A**) by 0.97 protons upon Ni(II) binding to Hpnl1 ligand and (**B**) by 1.00 protons upon Ni(II) binding to Hpnl2 ligand.

A plot of (ΔH_ITC_ + ΔH°_MB_) versus ΔH_HB_ for Ni(II)-Hpnl1 peptide is shown in Figure 6A and for Ni(II)-Hpnl2 peptide in Figure 6B. The number of protons transferred was determined. As the ΔH_ITC_ value decrease with decreasing buffer association enthalpy, ΔH_HB_ implies that the protons are transferred from the ligand to the buffer [13,26].

When a nickel ion binds to a certain amino acid side chain (e.g., histidine imidazole), it will displace a proton, but quantification of the number of protons is challenging because the environment surrounding the amino acid side chain can affect its pK_a_ and proton displacement cannot be reliably predicted from the pK_a_ values of free amino acids.

Protonation plots reveal (Figure 6) that the number of moles of the proton released by 1 mol of Hpnl1 and 1 mol of Hpnl2 during complexation of the Ni(II) ions equals 0.97 ± 0.13 and 1.00 ± 0.04, respectively.

These protons released upon Ni (II) binding could come from the deprotonation process of two possible ligand groups: a partially protonated histidine imidazole and the N-terminal amine that is expected to be mostly protonated at the pH of 7.4.

Mass spectrometry was also used to determine the stoichiometry of formed metal complexes. Mass spectrum for Ni(II)-Hpnl1 system is show in Figure S11. The three most intense peaks on the spectrum correspond to equimolar complexes with nickel(II) ions: [NiL]^2+^ (*m*/*z* = 759.79, monoisotopic mass at *z* = 2+), [NiL]^3+^ (*m*/*z* = 506.86, monoisotopic mass at *z* = 3+) and potassium to the Ni(II):Hpnl1 ion adduct—[NiL+K]^2+^ (*m*/*z* = 778.77, monoisotopic mass at *z* = 2+). Mass spectrum for Ni(II)-Hpnl2 system (Figure S12) shows very intense peaks for equimolar complexes with nickel [NiL]^2+^, [NiL]^3+^ and for free ligand [L]^3+^ with *m*/*z* = 636.23 (*z* = 2+), *m*/*z* = 424.49 (*z* = 3+) and *m*/*z* = 405.85 (*z* = 3+), respectively.

Mass spectra for the Ni(II)-Hpnl3 and Ni(II)-Hpnl3a are shown in Figures S13 and S14. The signal intensities for the nickel(II) complexes are much lower than in the previous systems, however, the formation of NiL species in the 1:1 (M:L) stoichiometry were observed. In case of Ni(II)-Hpnl3a system, the most intense peaks for the complex correspond to [NiL]^4+^ (*m/z* = 731.29, monoisotopic mass at *z* = 4+), [NiL]^5+^ (*m/z* = 585.24, monoisotopic mass at *z* = 5+) and [NiL]^3+^ (*m/z* = 974.72, monoisotopic mass at *z* = 3+). Similar specificity was observed for Ni(II)-Hpnl system, where peak at *m/z* = 747.80 (monoisotopic mass at *z* = 4+) corresponds to the most intense peak—[NiL]^4+^ and the next two species with similar intensity: [NiL]^5+^ and [NiL]^3+^ at *m/z* = 598.44 (*z* = 5+) and at *m/z* = 996.73 (*z* = 3+), respectively.

The other signals observed in the mass spectra are derived from differently ionized ligands, nickel(II)-ligand complexes, and their potassium and/or sodium ions adducts. The formation of complexes with a different stoichiometry than 1:1 was not observed. Each figure (Figures S11–S14) also shows the isotopic distribution of nickel(II)-peptide complex at chosen signals (usually the most intense ones) in the experimental and simulated spectra (A and B, respectively). The simulated and experimental data are in suitable agreement and confirm the formation of equimolar complexes (M:L, 1:1).

The visible Circular dichroism (CD) spectra show the formation of only one square-planar nickel complex for both ligands: Ni-Hpnl1 (Figure S15A) and Ni-Hpnl2 (Figure S17A). The involvement of imidazole nitrogens is confirmed by characteristic charge transfer transitions detected in CD spectra: N_im_→Ni(II), at about 260 nm. The resulting complexes with a 4N donor set have the maximum absorbance (λmax) of d–d bands at 410 nm and 478 nm for Hpnl1 peptide and 412 nm and 478 nm for Hpnl2 peptide [15,28]. There is no shift to the shorter or longer wavelengths with the addition of nickel ions up to two equivalents of Ni(II), which reveals that no additional metal ion is bound at this pH (7.4).

The UV-Vis spectra (Figures S16 and S18) additionally proved the formation of 4N Ni(II) complexes for both peptides. Far-ultraviolet CD spectroscopy has been applied to monitor changes in the secondary structure during complex formation. Some changes in the conformation have been shown for the Hpnl1 peptide after the addition of one equivalent of metal (S15B). They are not distinctive; however, there is some tendency toward the ß-sheet formation [29]. Far-ultraviolet CD spectra of Hpnl2 peptide reveal its random coil structure, which does not change after nickel addition up to 2 mols of metal per 1 mol of the peptide. For Hpnl3 and Hpnl3a peptides, because of aggregation phenomena, the spectra provided no clear information (data not shown).

It has been shown previously, depending on buffer composition and treatment of dithiothreitol (DTT), imidazole, and Ni(II), that some histidine-rich proteins form a range of multimeric complexes (Table 4). These proteins show an interesting phenomenon, named “gel shift” (unpredictable migration rate on SDS-PAGE against actual molecular weight (MW) formula, as shown in Table 4) [12]. The histidine- and glutamine-rich protein (Hpnl) was also shown, by gel-filtration chromatography and SDS-page, to form oligomers [11]. ITC studies of nickel-peptide analogs interactions, performed in buffers, seem to reflect the native behavior of the Hpn-like protein.

Table 4 also reveals that the differences in Ni(II) binding between these proteins are perhaps due to differences in the number of histidine residues available for interaction with the metal, and the number of glutamine residues has no impact on nickel binding in general.

## 3. Materials and Methods

All peptides (MAHHEQQQQQQA-NH_2_, MAHHEQQHQA-NH_2_, Ac-QHHHHHHAHHHHYYGGEHHHHNA-NH_2_, Ac-QHHHHHHAAHHHYYGGEHHHHNA-NH_2_) were purchased from KareBay Biochem (Monmouth Junction, NJ, USA) and were used as received (certified purity—98%). All solutions were prepared in ultra-pure deionized water (Polwater DL-3 (Krakow, Poland) equipped with UV lamp and 0.22 µm filter) with maximal conductivity of 0.06 µS/cm. Stock solutions were prepared using Mettler Toledo analytical balance with 0.01 mg precision. Solution of HCl (Chempur, Piekary Śląskie, Poland) in KCl (Chempur) was titrated by standardized 0.1 M NaOH (0.1 M NaOH concentrate from Sigma-Aldrich, Poznan, Poland). Carbonate-free NaOH solution (Sigma-Aldrich 0.1 M NaOH concentrate) was standardized by titration with potassium hydrogen phthalate (Sigma-Aldrich).

### 3.1. Isothermal Titration Calorimetry

Isothermal titration calorimetry (ITC) measurements were carried out at 25 °C and 37 °C on a MicroCal PEAQ titration calorimeter (Malvern, UK). All reagents, but KCl, were >99% pure and obtained from Sigma-Aldrich. The peptides were dissolved directly into buffer solution (TRIS, HEPES, or MOPS), whose pH was adjusted with NaOH or HCl to 7.4.

Metal stock solution (nickel(II) nitrate hexahydrate, 100 mM) was prepared in deionized water (maximal conductivity of 0.06 µS/cm) at low pH (~2) in acid-washed glass bottles. After stabilizing the instrument at 25 or 37 °C, 40 µL of a metal-buffer solution (1–2 mM) was used to titrate 200 µL of a peptide buffer solution, whose concentration was initially ten times smaller than that of the metal ion. Each titration consisted of 19 or 26 successive injections with an interval of 180–360 s between each aliquot (depending on the time needed for complete equilibration) and a stirring speed of 750 rpm, which was repeated at least three times for each temperature. The heat of a dilution from a corresponding control titration was subtracted before data fitting. An initial 0.4 µL injection was discarded from each data set to remove the effect of titrant diffusion across the syringe tip during the equilibration process. A CaCl_2_–EDTA titration was performed periodically for comparison to results obtained during the initial calibration of the instrument. The data were processed with MicroCal PEAQ-ITC Analysis Software. The one-site binding model provided the best-fit values of the stoichiometry (N), change in enthalpy (ΔH), and an equilibrium constant (K_d_).

### 3.2. Potentiometric Titration

The potentiometric titrations were performed using an automatic titrator system Omnis (Metrohm, Opacz-Kolonia, Poland) equipped with a combined glass electrode Biotrode^®^ with Idrolyte^®^ filling. The ionic strength was fixed at I = 0.1 M with KCl (Chempur). The combined glass electrode was calibrated as a hydrogen concentration probe by titrating known amounts of HCl (0.004 M) with carbonate-free NaOH solution (0.1 M). A stream of argon, pre-saturated with water vapor, passed over the surface of the solution cell, filled with 2.5 mL of investigated solution, thermostated at 25 ± 0.2 °C. For potentiometric measurements, the c_lig_ = 0.001 M, the metal to ligand ratios were 1:1 and 1:2.

The concentrations of peptides were determined from potentiometric titration of ligands. After careful calculation of concentration for each ligand, a sample from the same stock solution was taken for potentiometric measurements of the complex. The peptide solution was prepared freshly before measurements of ligand, in an amount sufficient to perform a titration of the ligand and then complex. The concentration of a stock solution of metal ions was determined by ITC titration with EDTA solution of known concentration. For complexes, around 150 data points were collected. The potentiometric data were analyzed with the Hyperquad 2013 program [42]. The distribution and competition diagram was computed with the HYSS program [43].

### 3.3. Mass Spectrometry

All Electrospray ionization-mass spectrometry (ESI-MS) experiments were performed on the LCMS-9030 qTOF Shimadzu (Shimadzu, Kyoto, Japan) device, equipped with a standard ESI source and the Nexera X2 system. Analysis was performed in the positive ion mode between 100 and 3000 *m*/*z*. LCMS-9030 parameters: nebulizing gas—nitrogen, nebulizing gas flow 3.0 L/min, drying gas flow—10 L/min, heating gas flow—10 L/min, interface temperature 300 °C, desolvation line temperature—400 °C, detector voltage—2.02 kV, interface voltage 4.0 kV, collision gas—argon, mobile phase (A) H_2_O + 0.1% HCOOH, (B) MeCN + 0.1% HCOOH, mobile phase total flow—0.3 mL/min. The injection volume was optimized depending on the intensity of the signals observed on the mass spectrum within the range of 0.1 to 1 μL. All obtained signals had a mass accuracy error in the range of 1 ppm. The concentration of peptide was 0.1 mM, and M:L molar ratio was 1:1. Samples were prepared in a mixture of water/methanol (50/50 *v*/*v*) at pH 7.40. All of the used solvents were of LCMS grade. The obtained data were analyzed by ACD/Spectrus Processor 220.2.0 (ACD/Labs, Toronto, ON, Canada).

### 3.4. UV-Vis and CD Spectroscopy

Circular dichroism (CD) measurements were obtained on a Jasco J-1500 CD spectrometer. Spectra were collected over the 800–230 nm range using quartz cuvettes with an optical path of 1 cm. The peptides and Ni(II) solutions were prepared in a MOPS buffer, pH 7.4. The concentration of each peptide was 0.5 mM. The spectra were taken after the addition of 0; 0.5; 1, and 2 equivalents of Ni (II). Absorption spectra were recorded in 20 mM HEPES buffer, 100 mM NaCl(pH 7.4) on a Cary 300 Bio spectrophotometer, in the range of 200–800 nm, using a quartz cuvette with an optical path of 1 cm. We could not obtain satisfactory results for Ni(II) binding to Hpnl3 and Hpnl3a due to the tendency to oligomerization of these peptides (opacity of solution). The titrations were carried out at 25 °C.

## 4. Conclusions

To summarize, the data presented here show that Hpn-like fragments are highly dynamic systems in terms of coordination chemistry. We compared Ni(II) binding between N-terminal and histidine-rich parts of Hpn-like and between N-terminal parts of the Hpnl proteins two *H. pylori* strains. Potentiometric, MS, UV-Vis, and CD spectroscopy data show that nickel ions bind to both N-terminal parts (peptides Hpnl1 and Hpnl2), forming stable, square-planar, 4N donor complexes with a 1:1 stoichiometry.

Post-hoc analysis of ITC experiments confirms the high affinity of these peptides toward nickel ions; however, the stoichiometry (N = 0.5) might suggest two peptides to one metal ion binding. In the ITC technique, the peptide at a given constant pH (here 7.4) was titrated by a metal solution prepared in this same buffer as a peptide. That differs from other techniques used in our studies.

Post-hoc analysis of ITC results also reveals that the number of moles of the proton released by 1 mol of Hpnl1 and 1 mol of Hpnl2 during complexation of the Ni(II) ions equals 0.97 ± 0.13 and 1.00 ± 0.04, respectively. Both N-terminal parts of Hpnl proteins have a higher affinity for Ni(II) than the His-rich part. Moreover, our results show that H29A is not central to metal binding in this (His-rich) part of the protein.

The ITC results also support the results of biochemical studies showing that histidine-rich proteins often form homo-dimers or oligomers in buffer solutions. In the cell, the precise nickel ion binding sites in proteins have both: albumin-like and poly-histidyl motifs, depending probably on the surrounding environment (other molecules, pH changes, etc.). The question remains: can the dissociation constants (K_d_) and the stoichiometry of the binding process in vitro be used to represent the conditions in vivo? If yes, which technique reveals the actual behavior of nickel complexation by Hpnl in *H. pylori* cells? The work brings up this phenomenon and paves the way for further studies that are necessary to solve these problems.

## Figures and Tables

**Table 1:** Experimental (conditional) thermodynamic values for Ni(II) binding to Hpnl1, Hpnl2, Hpnl3, and Hpnl3a peptides from ITC measurements in three different buffers, at 37 °C.

Ligand	Data (Units)	TRIS	HEPES	MOPS
Hpnl1	N_ITC_	0.51 ± 0.01	0.43 ± 0.01	0.54 ± 0.01
	K_dITC_ (μM)	3.24 ± 0.37	3.16 ± 0.66	2.01 ± 0.24
	ΔH_ITC_ (kcal/mol)	−8.51 ± 0.18	−3.31 ± 0.12	−2.86 ± 0.06
	−TΔS_ITC_ (kcal/mol)	0.70	−4.50	−5.23
Hpnl2	N_ITC_	0.46 ± 0.003	0.43 ± 0.01	0.59 ± 0.01
	K_dITC_ (μM)	2.44 ± 0.17	2.45 ± 0.37	2.12 ± 0.33
	ΔH_ITC_ (kcal/mol)	−13.2 ± 0.14	−3.1 ± 0.08	−2.83 ± 0.07
	−TΔS_ITC_ (kcal/mol)	5.28	−4.86	−5.22
Hpnl3	N_ITC_	0.51 ± 0.01	0.45 ± 0.01	0.46 ± 0.01
	K_dITC_ (μM)	4.64 ± 0.68	7.8 ± 0.54	8.3 ± 0.85
	ΔH_ITC_ (kcal/mol)	−29.4 ± 0.94	−19.4 ± 0.4	−15.2 ± 0.44
	−TΔS_ITC_ (kcal/mol)	21.80	12.10	8.00
Hpnl3a	N_ITC_	0.43 ± 0.01	0.55 ± 0.01	0.47 ± 0.01
	K_dITC_ (μM)	1.03 ± 0.2	4.9 ± 0.06	2.53 ± 0.34
	ΔH_ITC_ (kcal/mol)	−32.4 ± 0.75	−23.9 ± 0.77	−19.8 ± 0.53
	−TΔS_ITC_ (kcal/mol)	23.90	16.40	11.90

**Table 2:** Experimental (conditional) thermodynamic values for Ni(II) binding to Hpnl1, Hpnl2, Hpnl3, and Hpnl3a peptides from ITC measurements in three different buffers, at 25 °C.

Ligand	Data (Units)	TRIS	HEPES	MOPS
Hpnl1	N_ITC_	0.45 ± 0.01	0.45 ± 0.02	0.54 ± 0.01
	K_dITC_ (μM)	11.4 ± 2.33	3.79 ± 0.57	5.09 ± 0.66
	ΔH_ITC_ (kcal/mol)	−12.1 ± 0.7	−3.96 ± 0.13	−4.26 ± 0.11
	−TΔS_ITC_ (kcal/mol)	5.39	−3.44	−2.97
Hpnl2	N_ITC_	0.48 ± 0.01	0.59 ± 0.03	0.51 ± 0.01
	K_dITC_ (μM)	7.98 ± 1.22	5.74 ± 1.06	4.12 ± 0.52
	ΔH_ITC_ (kcal/mol)	−11.70 ± 0.46	−1.86 ± 0.17	−4.44 ± 0.12
	−TΔS_ITC_ (kcal/mol)	4.74	−5.29	−2.92
Hpnl3	N_ITC_	0.94 ± 0.01	0.41 ± 0.01	0.45 ± 0.01
	K_dITC_ (μM)	9.47 ± 1.22	6.41 ± 0.55	5.27 ± 0.55
	ΔH_ITC_ (kcal/mol)	−27.5 ± 0.98	−15.6 ± 0.38	−11.4 ± 0.27
	−TΔS_ITC_ (kcal/mol)	20.70	8.53	4.22
Hpnl3a	N_ITC_	0.93 ± 0.01	0.44 ± 0.01	0.49 ± 0.01
	K_dITC_ (μM)	1.06 ± 0.25	4.03 ± 0.86	2.11 ± 0.43
	ΔH_ITC_ (kcal/mol)	−31.3 ± 0.99	−22.8 ± 1.01	−20.7 ± 0.65
	−TΔS_ITC_ (kcal/mol)	23.10	15.40	12.90

**Table 3:** Protonation constants of MAHHEQQQQQQA-NH_2_ and MAHHEQQHQA-NH_2_ peptides. The concentration was 0.001 M for each peptide. Titrations were carried out over the pH range 2–11 at T = 25 °C in an aqueous solution with 4 mM HCl and 0.1 M KCl.

Species	MAHHEQQQQQQA-NH_2_ (Hpnl1)		MAHHEQQHQA-NH_2_ (Hpnl2)	
	logβ	logK	logβ	logK
LH	7.53 (1)	7.53	7.58 (1)	7.58
LH_2_	14.07 (1)	6.54	14.35 (1)	6.77
LH_3_	20.04 (1)	5.97	20.63 (1)	6.28
LH_4_	24.11 (2)	4.07	26.36 (1)	5.73
LH_5_	-	-	30.31 (1)	3.95

**Table 4:** Histidine-rich proteins and their properties.

Protein	Bacterium Name	No. of Histidine (H) Residues Per Histidine-Rich Domain	No. of Glutamine (Q) Per Protein Monomer	No.of Ni(II) Ions Per Monomer	K_d_ (µM)	Formula MW (kDa)	Apparent MW (kDa)	References
HypB	*Bradyrhizobium japonicum*	24/39	5	9	2.3	32.5	38, 78(dimer)	[30]
Hyp B	*Rhizobium leguminosarum*	17/32	6	4	2.5	32	39	[1,31]
UreE	*Klebsiella aerogenes*	10/15	5	3	9.6	17.5	35 (dimer)	[32,33]
CooJ	*Rhodospirillum rubrum*	16/34	1	4	4.3	19	39 (dimer)	[34]
SlyD	*Escherichia coli*	15/50	6	3–7	2	20.8	25	[35,36]
HspA	*Helicobacter* *pylori*	8/27	1	2	1.8	13	13	[37,38,39]
Hpn	*Helicobacter* *pylori*	28/60	2	5–6	7.1	7	7, 14, 20, 70, 136, 230…	[40,41]
Hpn-like (Hpnl) strain 11637	*Helicobacter* *pylori*	14/23	31	2	3.8	9	18 (dimer), 28, 36, 47, 201	[1,11]

## Data Availability

All the data supporting the conclusions of this article are provided within the article and in its additional files. All data and materials are available upon reasonable request from the corresponding authors.

## Supplementary Materials

The following are available online at https://www.mdpi.com/article/10.3390/ijms222413210/s1.
