# Supplementary material for: A Comparative Study on Nickel Binding to Hpn-like Polypeptides from Two Helicobacter pylori Strains
Source: Int J Mol Sci. 2021 Dec 8;22(24):13210. doi: 10.3390/ijms222413210 (PMC8704837; doi:10.3390/ijms222413210)
Supplement: Supplementary file 1 [file ijms-22-13210-s001.zip › ijms-1377912-supplementary.pdf]

# A comparative study on nickel binding to Hpn-like polipeptides from two *Helicobacter pylori* strains.

*D. Witkowska, A. Szebesczyk, A.Watly, M.Rowińska-Żyrek, M.Braczkowski*

## Supplementary materials

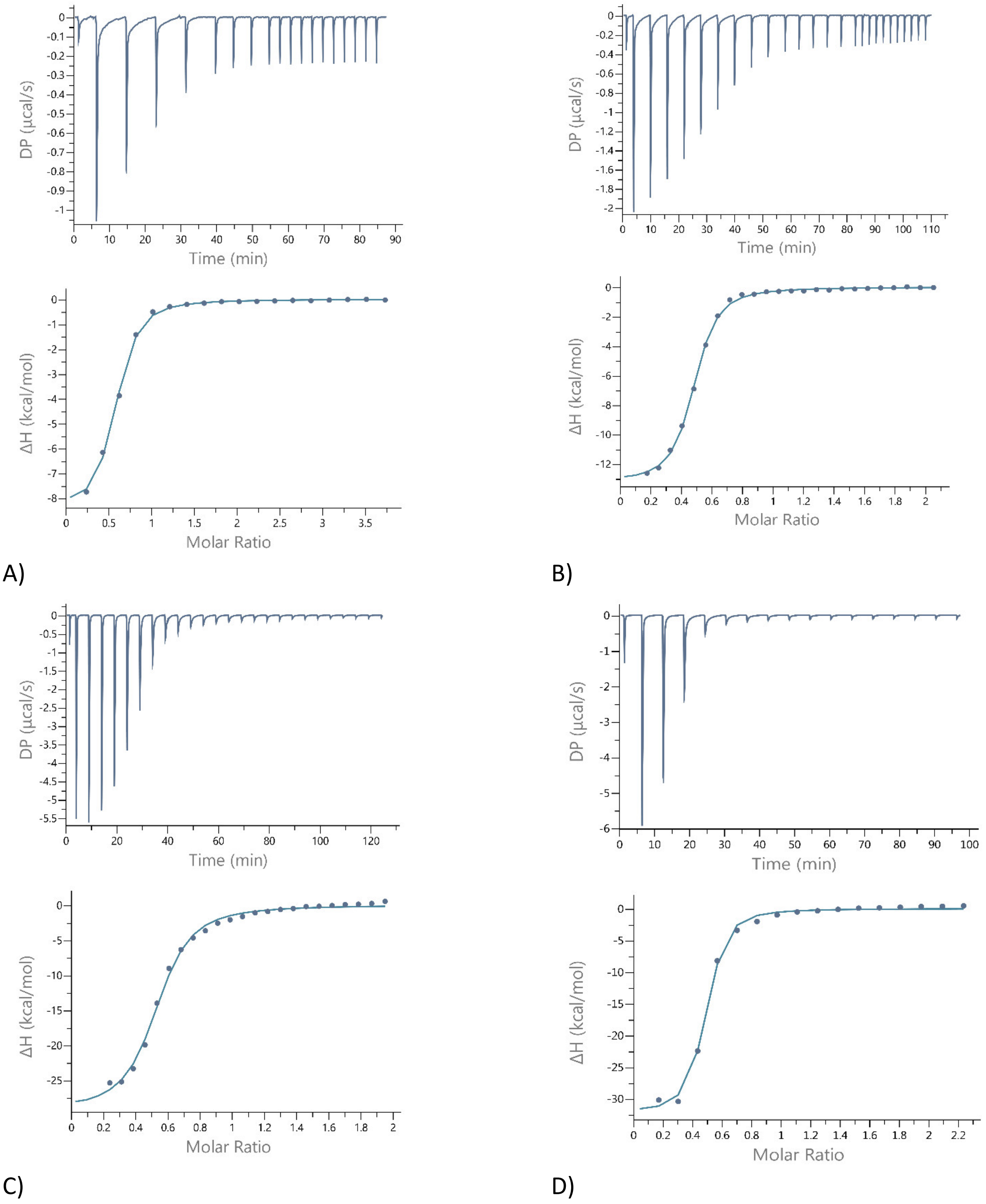


**Figure S1**. The best-fit ITC data for the 37 ˚C titration of 2mM Ni(II) into (**A**) Hpnl1, (B) Hpnl2, (**C**) Hpnl3, (**D**) Hpnl3a peptides in 20mM TRIS-HCl buffer + 500mM NaCl, pH 7.40. The peptides concentration was within the range of 100-200 µM.

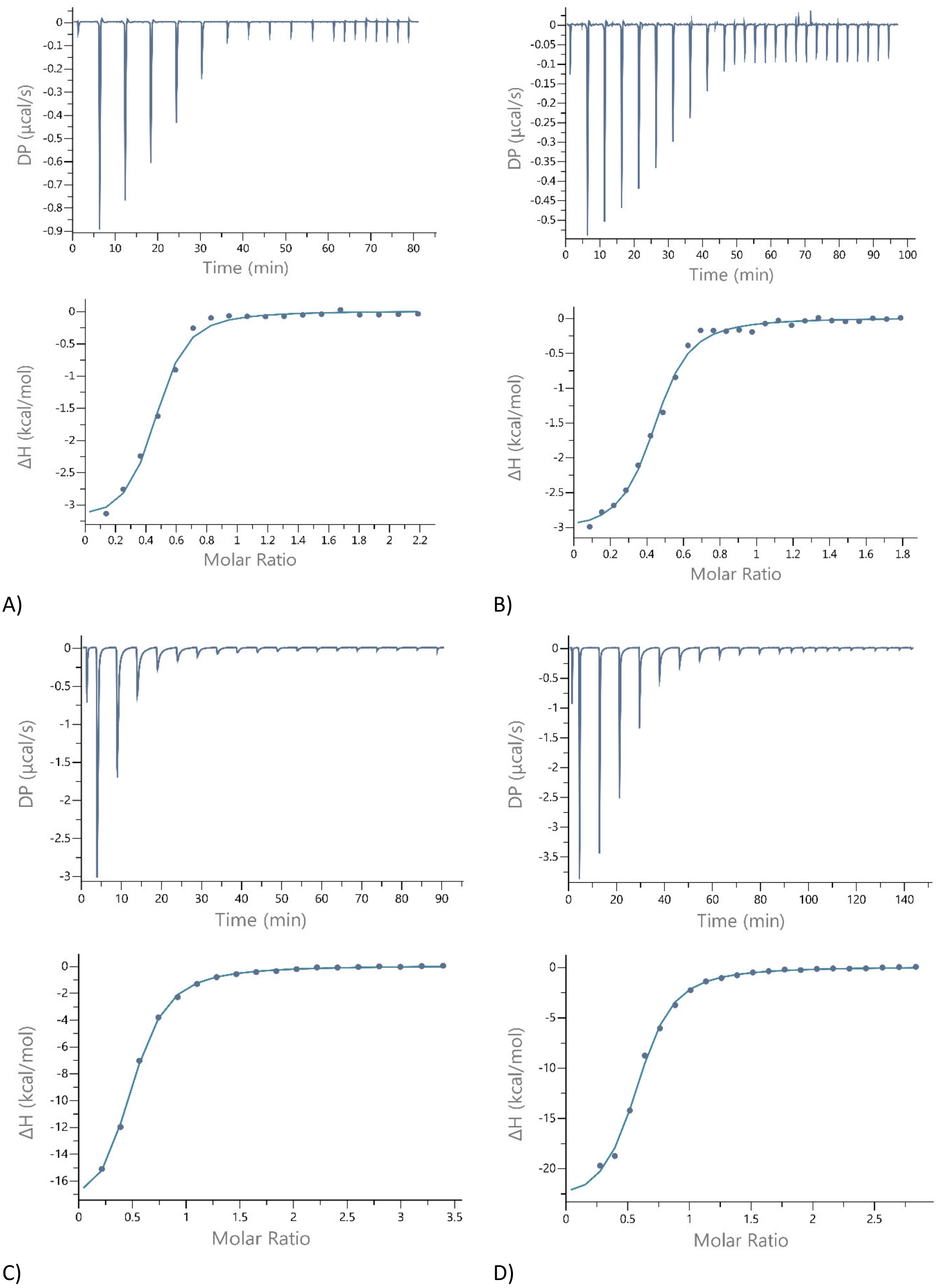


**Figure S2**. The best-fit ITC data for the 37 ˚C titration of 1-2mM Ni(II) into (**A**) Hpnl1, (**B**) Hpnl2, (**C**) Hpnl3, (**D**) Hpnl3a peptides in 20mM HEPES buffer + 100mM NaCl, pH 7.40. The peptides concentration was within the range of 109-128 µM.

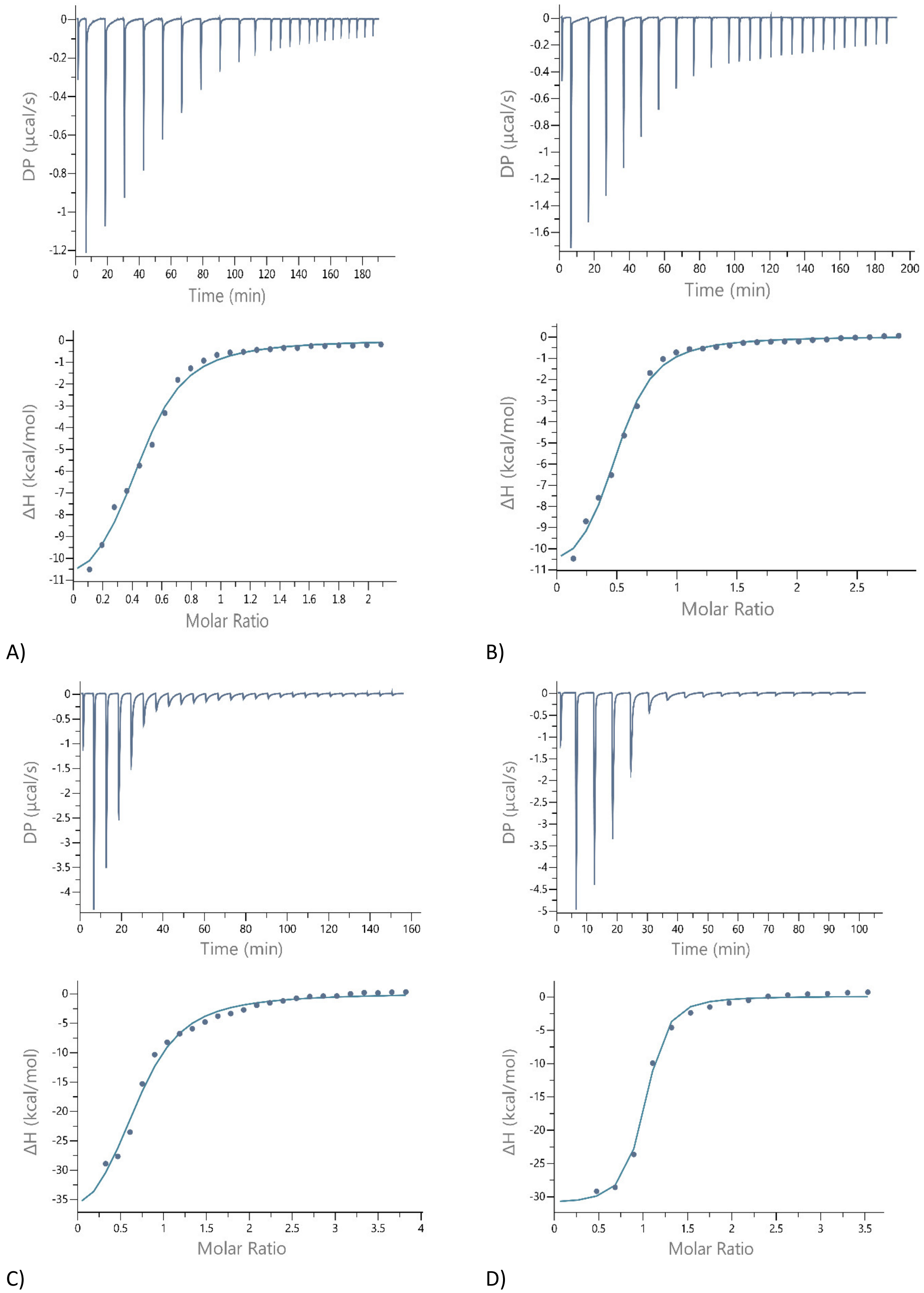


**Figure S3**. The best-fit ITC data for the 25 ˚C titration of 2mM Ni(II) into (**A**) Hpnl1, (**B**) Hpnl2, (**C**) Hpnl3, (**D**) Hpnl3a peptides in 20mM TRIS-HCl buffer + 500mM NaCl, pH 7.40. The peptides concentration was within the range of 106-171 µM.

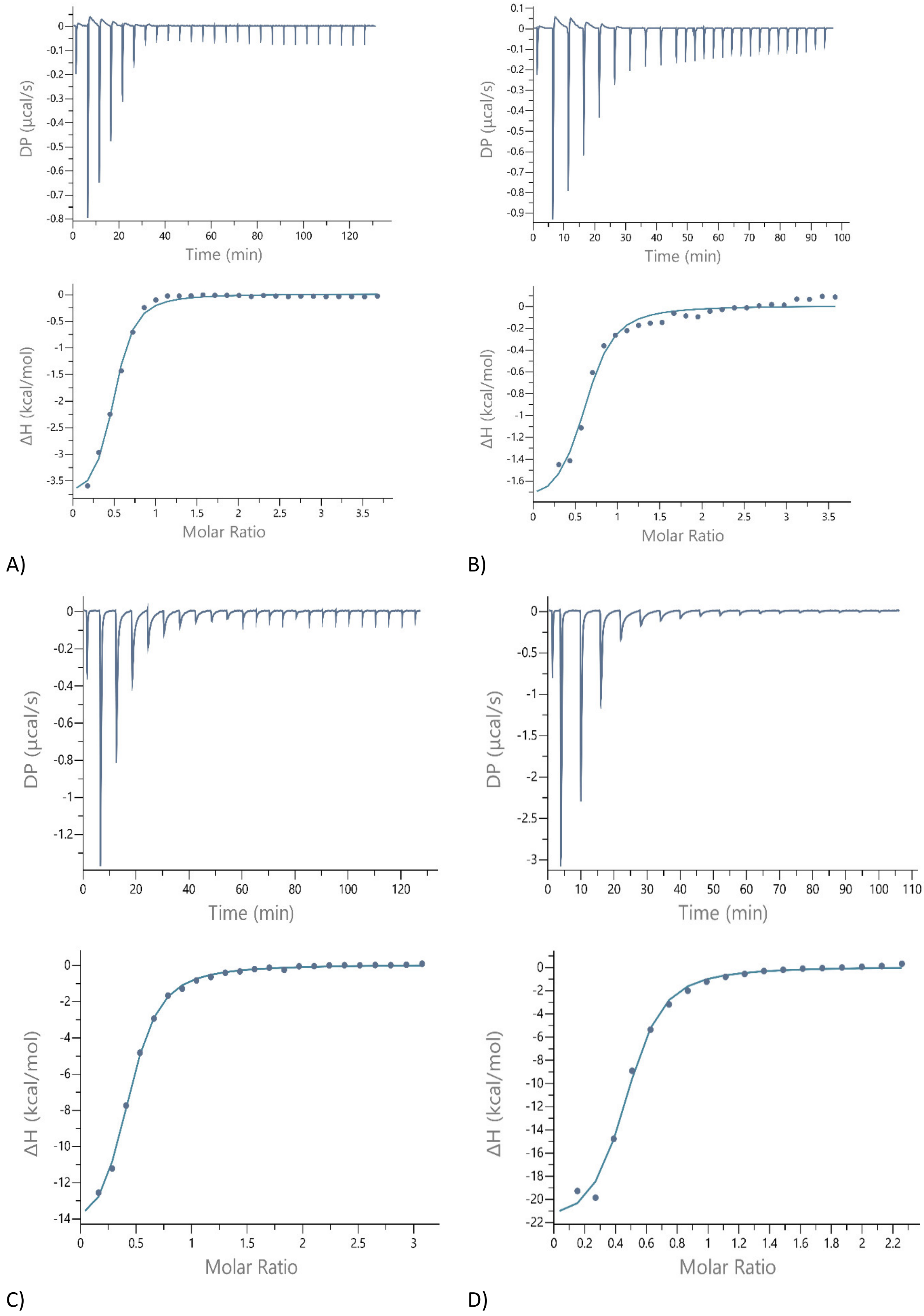


**Figure S4**. The best-fit ITC data for the 25 ˚C titration of 2mM Ni(II) into (**A**) Hpnl1, (**B**) Hpnl2, (**C**) Hpnl3, (**D**) Hpnl3a peptides in 20mM HEPES buffer + 100mM NaCl, pH 7.40. The peptides concentration was within the range of 109-122 µM.

**Table S1**. Potentiometric data for Ni (II) complexes with Hpnl1 (MAHHEQQQQQQA-$NH_2$) and Hpnl2 (MAHHEQQHQA-$NH_2$). Titrations were carried out over the pH range 2–11 at T = 25 ˚C in an aqueous solution with 4 mM HCl and 0.1 M KCl. The peptide concentration was 0.001 M and the Ni(II)-to-peptide ratios were 1:1, and 1:2.

| Species | Hpnl1 MAHHEQQQQQQA-$NH_2$ | | | | Hpnl2 MAHHEQQHQA-$NH_2$ | | | |
|---|---|---|---|---|---|---|---|---|
| | Ni:L 1:1 | | Ni:L 1:2 | | Ni:L 1:1 | | Ni:L 1:2 | |
| | logβ | logK | logβ | logK | logβ | logK | logβ | logK |
| $NiLH_2$ | 17.61 (7) | | 18.31 (8) | | | | | |
| NiLH | | | | | 12.15 (5) | | 12.23 (5) | |
| NiL | 5.76 (7) | | 6.58 (6) | | | | | |
| $NiLH_{-1}$ | | | | | -0.24 (3) | | -0.81 (4) | |
| $NiLH_{-2}$ | -6.64 (9) | | -6.61 (8) | | -7.46 (6) | 7.22 | -8.05 (9) | 7.24 |
| $NiLH_{-3}$ | | | -13.72 (9) | 7.11 | | | | |

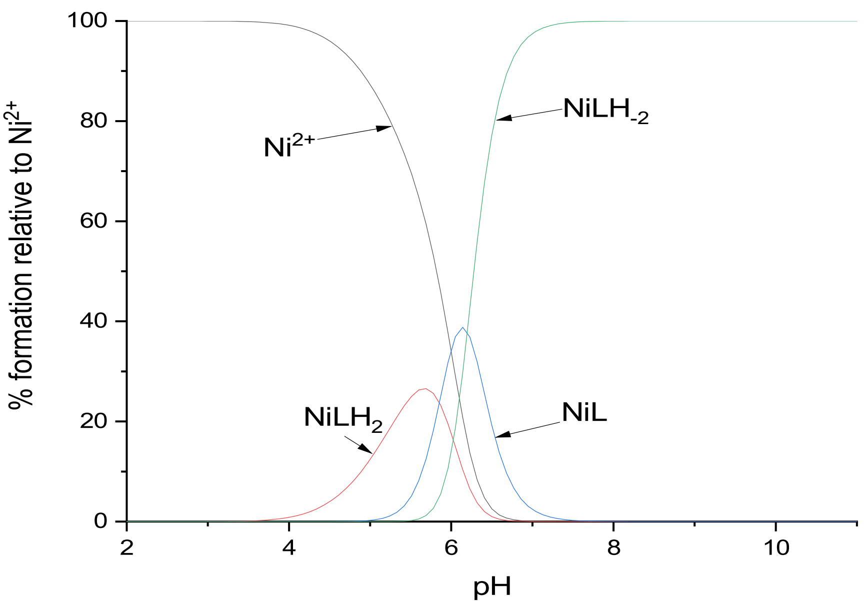


**Figure S5**. Species distribution profiles for Ni(II) complexes of MAHHEQQQQQQA-$NH_2$. Metal to ligand ratio = 1:1.

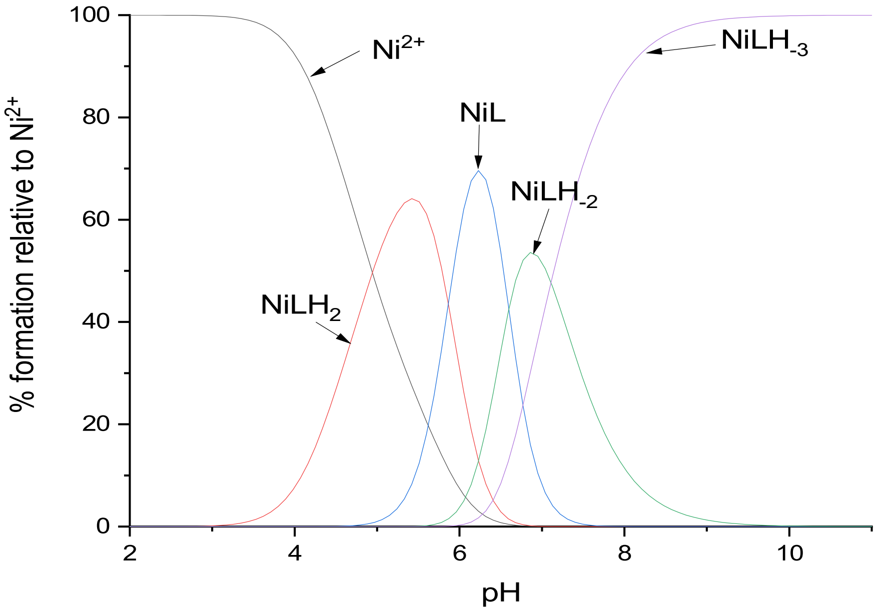


**Figure S6.** Species distribution profiles for Ni(II) complexes of MAHHEQQQQQQA-$NH_2$. Metal to ligand ratio = 1:2.

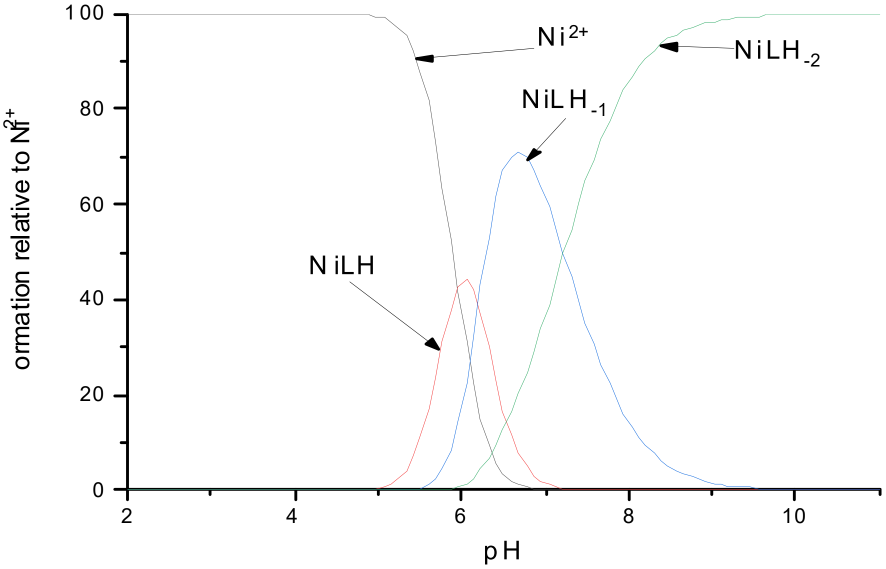


**Figure S7**. Species distribution profiles for Ni(II) complexes of MAHHEQQHQA-$NH_2$. Metal to ligand ratio = 1:1.

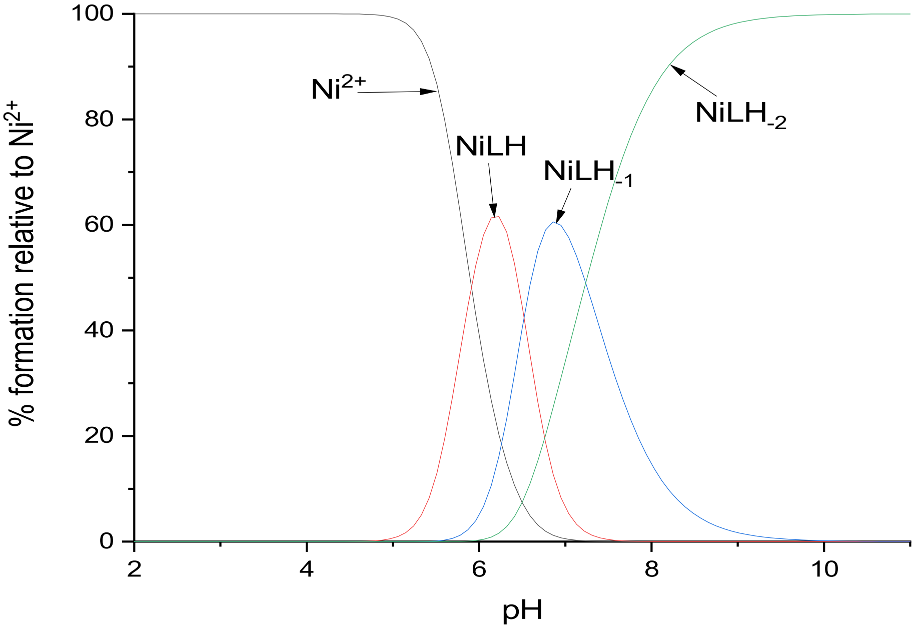


**Figure S8**. Species distribution profiles for Ni(II) complexes of MAHHEQQHQA-$NH_2$. Metal to ligand ratio = 1:2.

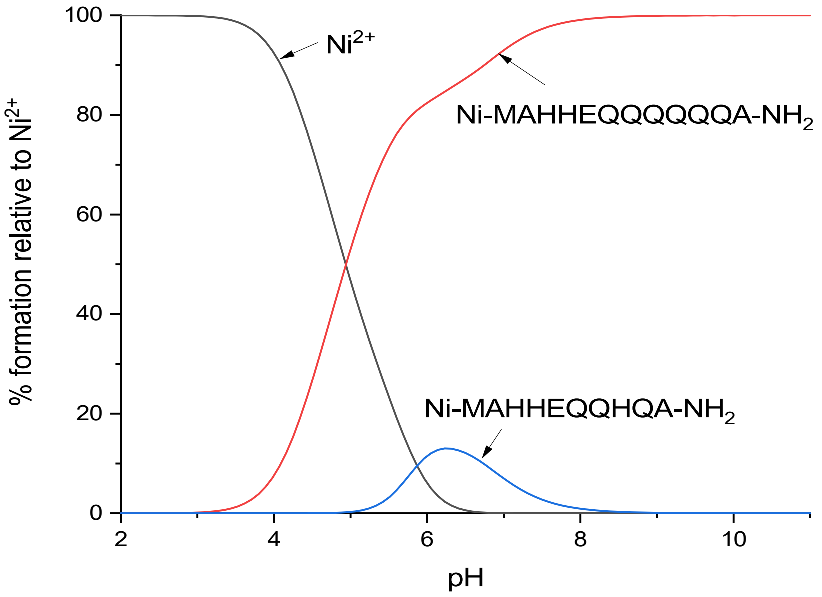


**Figure S9**. Competition plot for MAHHEQQQQQQA-$NH_2$, MAHHEQQHQA-$NH_2$, and Ni(II) showing the relative amount of each complex at different pH values for the hypothetical situation in which equimolar amounts of the two species are mixed. [Ni(II)]=0.0005 M. Ni(II) to ligand ratio of 0.5:1:1.

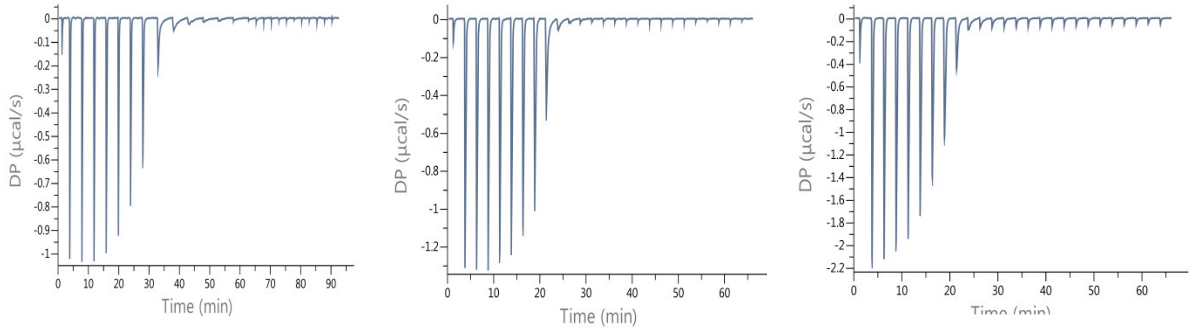


A B C

**Figure S10**. ITC traces of 1mM Ni(II) to 0,1 mM EDTA titration in (**A**) HEPES, (**B**) MOPS, and (**C**) TRIS buffer, pH 7.4. The average $\Delta H_{ITC}$ for at least 2 best fit titration was -7.94±0.2 kcal/mol; -9.73±0.21 kcal/mol; and -17.4±0.16 kcal/mol for HEPES, MOPS, and TRIS buffer, respectively.

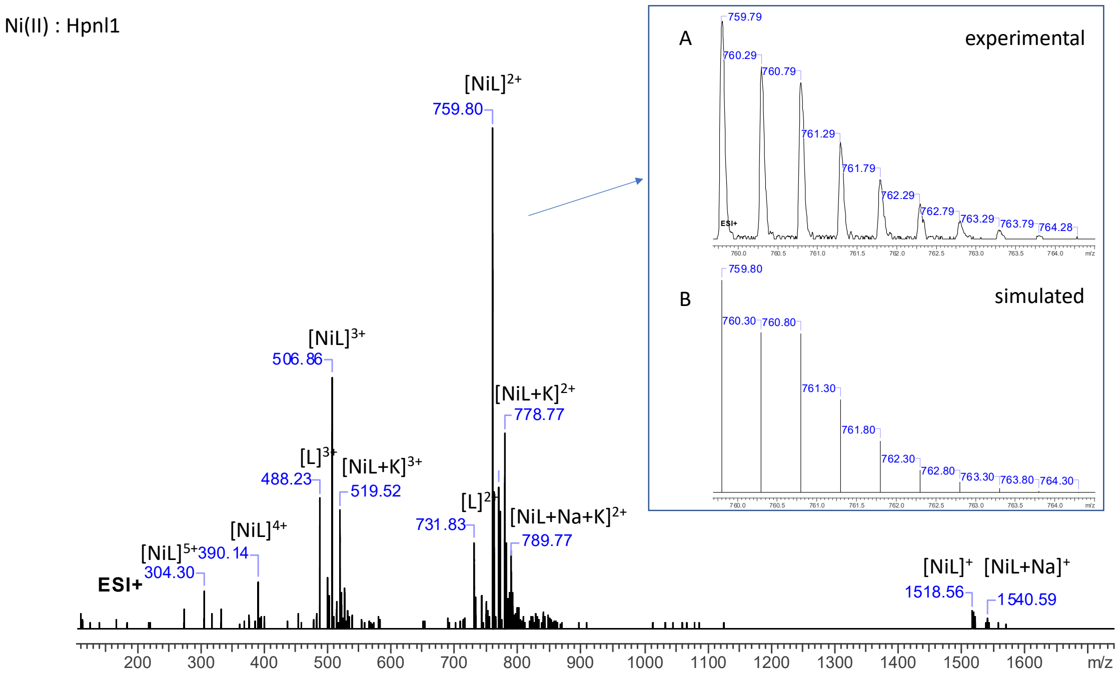


**Figure S11**. ESI positive mass spectrum (ESI-MS) for the Ni(II):Hpnl1 system with experimental (**A**) and simulated (**B**) spectra for chosen signal, $[NiL]^{2+}$. Metal:ligand in a 1:1 stoichiometry, where $[ligand]_{tot}$= 0.1 mM. Measurements were prepared in water/methanol (50/50, v/v) mixture at pH 7.40.

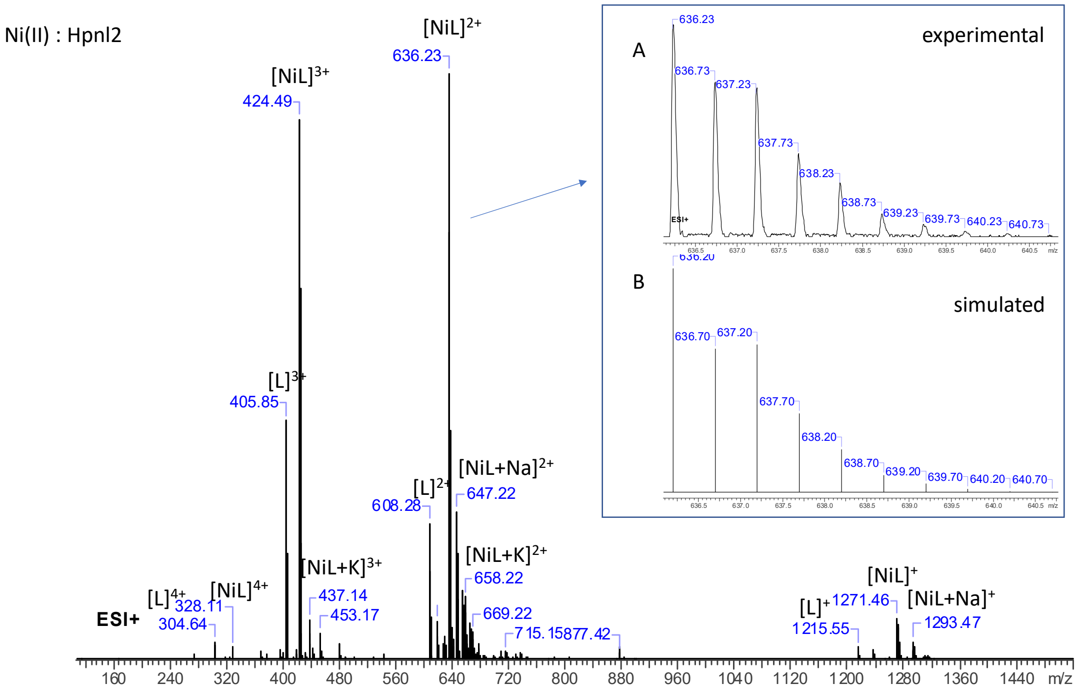


**Figure S12**. ESI positive mass spectrum (ESI-MS) for the Ni(II):Hpnl2 system with experimental (**A**) and simulated (**B**) spectra for chosen signal, $[NiL]^{2+}$. Metal:ligand in a 1:1 stoichiometry, where $[ligand]_{tot}$= 0.1 mM. Measurements were prepared in water/methanol (50/50, v/v) mixture at pH 7.40.

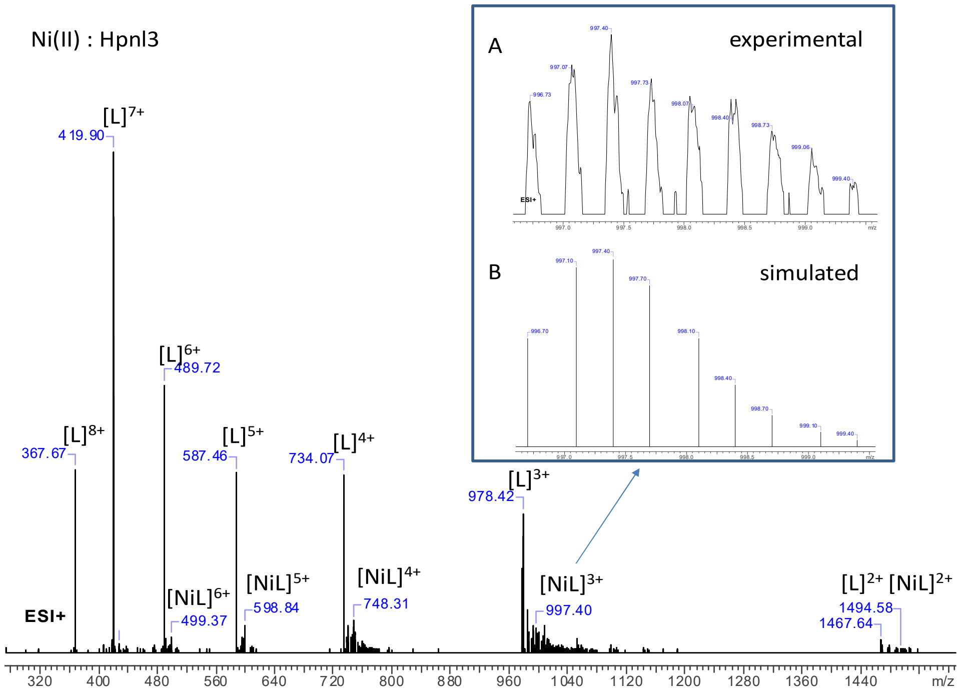


**Figure S13**. ESI positive mass spectrum (ESI-MS) for the Ni(II):Hpnl3 system with experimental (**A**) and simulated (**B**) spectra for chosen signal, $[NiL]^{3+}$. Metal:ligand in a 1:1 stoichiometry, where $[ligand]_{tot}$ = 0.1 mM. Measurements were prepared in water/methanol (50/50, v/v) mixture at pH 7.40.

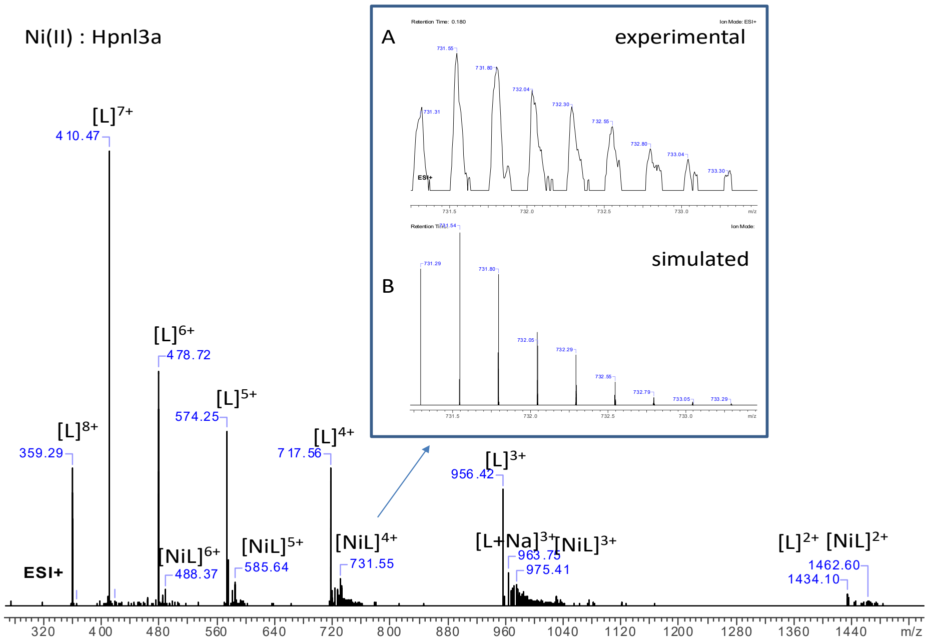


**Figure S14**. ESI positive mass spectrum (ESI-MS) for the Ni(II):Hpnl3a system with experimental (**A**) and simulated (**B**) spectra for chosen signal, $[NiL]^{3+}$. Metal:ligand in a 1:1 stoichiometry, where $[ligand]_{tot}$= 0.1 mM. Measurements were prepared in water/methanol (50/50, v/v) mixture at pH 7.40.

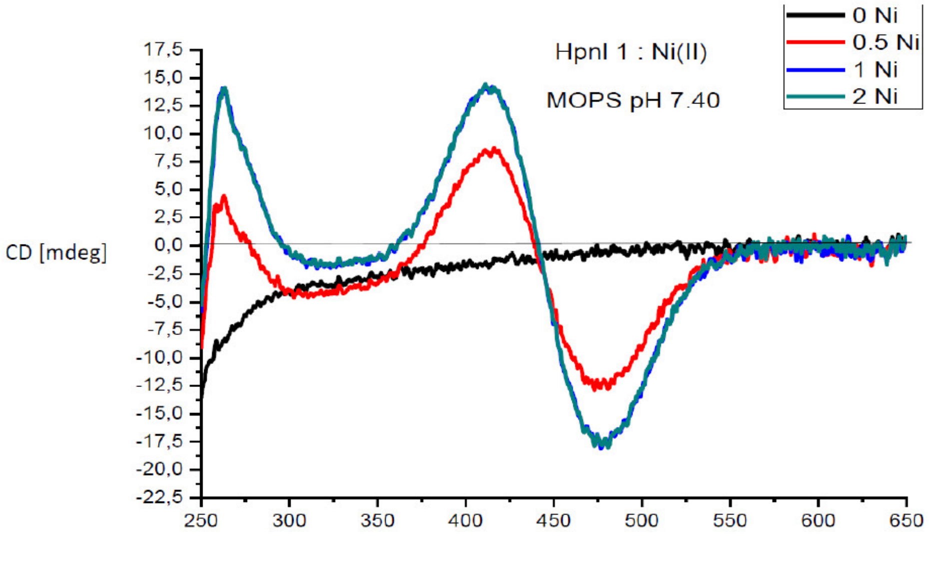


A) nm

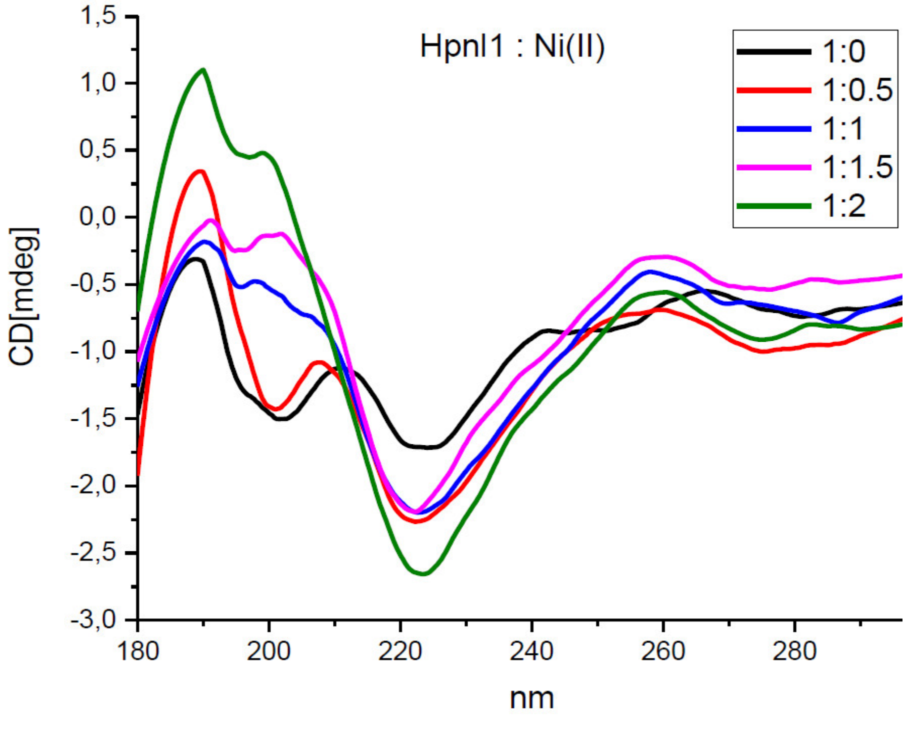


B)

**Figure S15**. (**A**) The visible CD and (**B**) far-UV CD spectra of Ni(II) complexes of MAHHEQQQQQQA-NH2 (Hpnl1) peptide, pH 7,4.

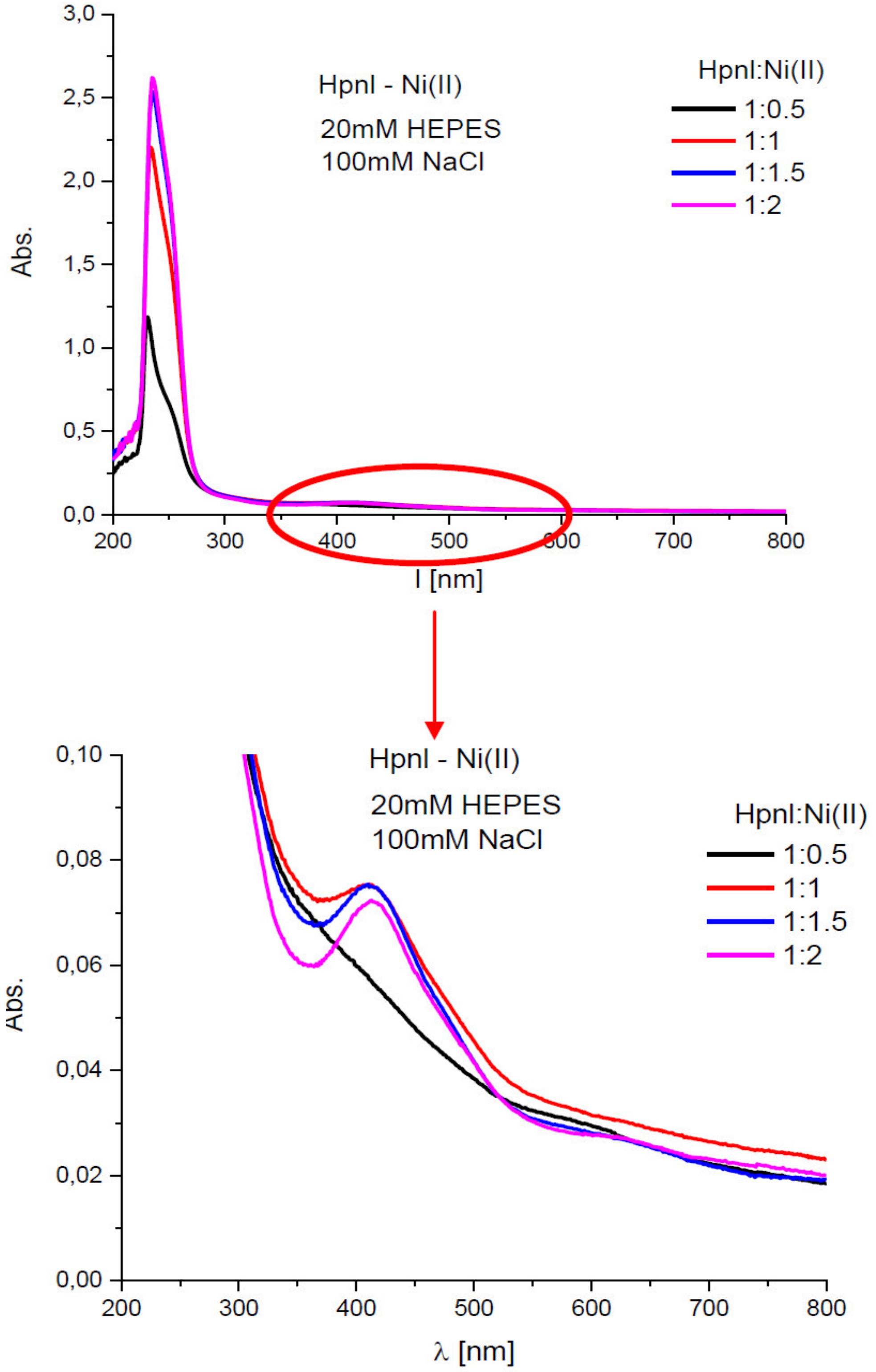


**Figure S16**. UV-Vis spectra of Ni(II) complexes of MAHHEQQQQQQA-NH2 (Hpnl1) peptide, pH 7,4.

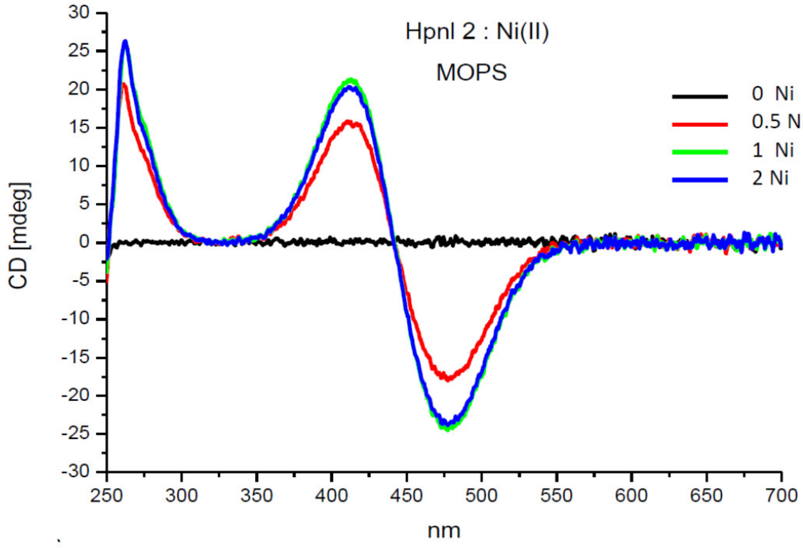


A)

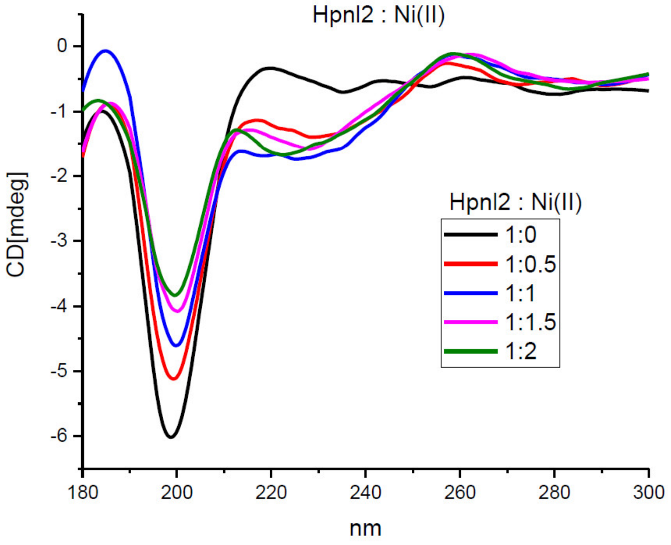


B)

**Figure S17**. (**A**) The visible CD and (**B**) far-UV CD spectra of Ni(II) complexes of Hpnl 2 (MAHHEQQHQA-NH2) peptide, pH 7,4.

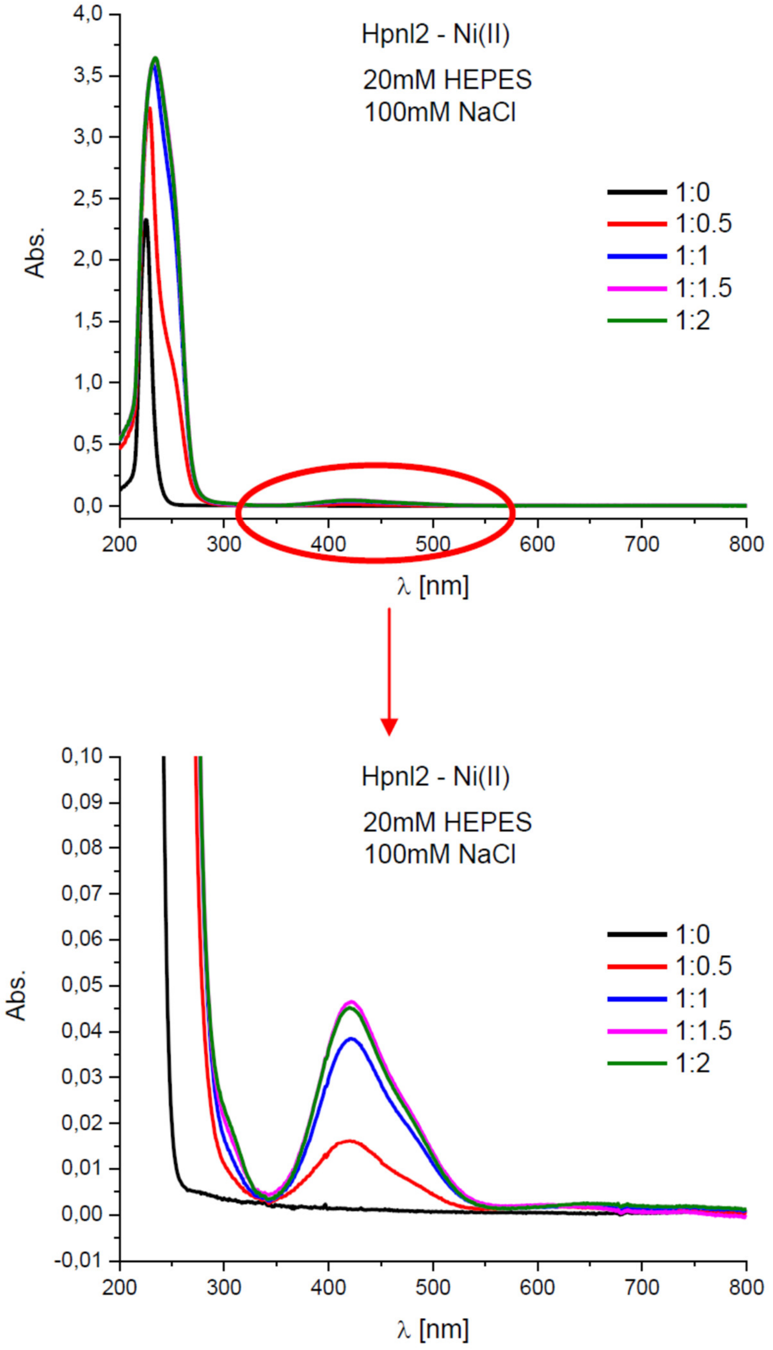


**Figure S18**. UV-Vis spectra of Ni(II) complexes of Hpnl 2 (MAHHEQQHQA-NH2) peptide, pH 7,4.
